# Ethics of Clinical Science in a Public Health Emergency: Drug Discovery at the Bedside

**DOI:** 10.1080/15265161.2013.813597

**Published:** 2013-08-16

**Authors:** Sarah J. L. Edwards

**Affiliations:** ^a^ University College London

**Keywords:** research ethics, public health

## Abstract

Clinical research under the usual regulatory constraints may be difficult or even impossible in a public health emergency. Regulators must seek to strike a good balance in granting as wide therapeutic access to new drugs as possible at the same time as gathering sound evidence of safety and effectiveness. To inform current policy, I reexamine the philosophical rationale for restricting new medicines to clinical trials, at any stage and for any population of patients (which resides in the precautionary principle), to show that its objective to protect public health, now or in the future, could soon be defeated in a pandemic. Providing wider therapeutic access and coordinating observations and natural experiments, including service delivery by cluster (wedged cluster trials), may provide such a balance. However, there are important questions of fairness to resolve before any such research can proceed.

Most discussion of ethics in a public health emergency focuses on questions of distribution of available resources, and the main aim of current emergency planning is to establish a priority list to help decide who should get any available treatments first (WHO 2007). With a couple of notable exceptions (London 2009; WHO 2009), there is little consideration paid to the process(es) by which we might gather scientifically robust evidence to support these distributive decisions. As a corollary of this neglect, we cannot even be sure whether these first “lucky” recipients of new treatments count technically as “research subjects” in the same way as people enrolled in a formal Phase I trial. If patients receiving new treatments are not, strictly speaking, also research subjects, they cannot expect to enjoy the same level of protective oversight that regulators provide within the tightly formalized systems and institutional structures that govern research (McRae et al. 2011; WHO 2009). In any case, having decided what counts as formal research, and thus what formal protection the systems of research regulation can offer, running a program of clinical science takes a considerable amount of logistic organization and time in order to execute it efficiently and effectively.

Regulation of investigational new drugs often means that access to them is tightly restricted to a lengthy research program unless and until there is sufficient evidence that they are safe and efficacious. Such research evidence is usually sought through a series of three phases of clinical research that might test any given drug for its effects in any one condition. However, in some cases, the process by which we approach clinical science and its regulation must adapt to the circumstances in question. For example, following the rise of HIV/AIDS in the 1980s, the Food and Drug Administration (FDA) introduced new rules, apparently to widen therapeutic access to investigational new drugs (National Academy of Sciences 1991). While the case of HIV provides a precedent for expanding access to investigational treatments to some extent, many would welcome ways to widen access even further without also compromising on research evidence (National Academy of Sciences 1991).^1^ My task in this article is to find an acceptable balance, for regulators, between providing very ill patients with new therapeutic treatments and pursuing scientific evidence of safety and effectiveness in the most challenging circumstances imaginable, namely, a pandemic. Given that the scope of scientific inquiry is greatly compromised by these circumstances, notwithstanding the conclusion of the World Health Organization (WHO) report in 2009, there might well be special questions of ethics and regulation relating to this sort of research. Some, more standard, types of research might not be feasible or they might be incompatible with other nonpharmaceutical measures such as geographical containment, or even quarantine, to curtail the spread of a communicable disease. There are thus many questions associated with how clinical science might ethically be conducted in a pandemic, such as when consent might be waived in an emergency, but here I wish to focus on what I consider to be the prior issue of how regulators should strike a good balance between therapeutic access and scientific endeavor.1. It is useful to note that the widest “therapeutic access” to new medicines, which might mean abandoning the quest for robust research evidence, does not imply that regulation of investigational new drugs must also be abandoned. Conversely, it might also be possible to collect robust research data in a completely unregulated market for medicines. In this article, I am interested in showing how regulators might consider the balance between research and practice in a public health crisis.


It is worth noting that, to some extent and in some cases such as pandemic flu, it might be possible to discover new compounds and tests new drugs under tightly controlled and managed circumstances (as current rules require).^2^ However, pandemic flu is not the only public health emergency we could encounter. We could encounter a rather more unexpected threat, from a pathogen that has never been seen before, for which we have no known effective treatment, and against which we have no way of forearming ourselves. A good example is provided by the severe acute respiratory syndrome (SARS) outbreak in 2002–2003. SARS almost became a pandemic: The WHO 2004 report lists 8,096 known infected cases and 774 deaths. In the case of SARS, because the pathogen was new, we had very little time to prepare against it and the disease was already rampant before we had any idea what it was, let alone had any effective treatments for it.^3^ More recently, a new SARS-like virus of the coronavirus family has been discovered in Saudi Arabia in 2012, although it is not thought to be spread easily, so travel restrictions have not (yet) been imposed (BBC 2012).I thank James Wilson, whose teaching notes filled gaps in my knowledge of pandemics.
In addition, the fact that the Chinese government failed to notify the WHO about the spreading virus until it was much too late added to the problems.


To set the scene in more depth, in the second section of this article I rehearse current regulation of new health technologies in general terms and show that clinical research under existing regulatory constraints (including the FDA schemes of expedited development and parallel track) may be difficult or even impossible in a public health emergency. In the third section, I reexamine the rationale for restricting new medicines to clinical trials (at any stage of the development process and for any patient population) to show that its objective to protect public health, now or in the future, could soon be defeated in a pandemic. Furthermore, I suggest that the method used to achieve such a public health objective (which places a burden of scientific proof on researchers to gather evidence of safety, and in most cases efficacy, *before* making a new treatment widely available) may require judicial application. However, in the fourth section, I discuss why some regulation of clinical research, despite the extreme circumstances of a pandemic, is ethically required. In light of these points, in the fifth section, I explore how regulation might offer a good balance between the need to widen therapeutic access to new treatments and the need to gain scientific information. I suggest that the use of research designs such as cluster randomized controlled trials (CRCTs) might be suitable in this instance. Randomized cluster trials can be used to introduce a new therapy in a stepwise fashion to the population at large without restricting it first to a series of trials and only then distributing it more widely. Those clusters waiting for the therapy act as controls in the meantime. The distinction between research and practice thus falls away, at least for the purposes of therapeutic access. However, as I show in the sixth section, there are important moral limits to the use of alternative research designs, as described earlier, especially in such a crisis. These must be carefully considered before mounting clinical research along what may become politically divisive lines. These limitations, however, might not be prohibitive in the end, but serious attention should, nonetheless, be paid to how social conflict and attendant health inequalities could be mitigated by their use.

## EXISTING REGULATORY FRAMEWORK AND ITS LIMITS

Potential drug candidates, once identified, need to be clinically evaluated. Before any new compound may legally be “tested” in a human being, however, regulators generally ask to see extensive preclinical information which includes safety and dose testing on animals.^4^ Many countries regulate new health technologies thereafter by restricting use of them to clinical trials (to check that they are safe and efficacious) before licensing them for sale on the open market. For example, in the United States the Food and Drug Administration (FDA) and in the United Kingdom the Medicines and Health Care Products Regulatory Authority (MHRA) require all new drugs, and many new medical devices, to undergo a series of clinical trials before they will consider granting a marketing license. Phase I is commonly a relatively small study, usually involving healthy volunteers, to determine which dose of a new drug is tolerated by the human body. Only when the new drug is known to have severe adverse side effects, as in the case of chemotherapeutic agents, are patients recruited, and even then, only when all other treatment options have been exhausted. Patients are usually recruited at Phase II (when the first tests of efficacy are run) and then again at Phase III (in a much larger cohort to gain statistically more precise estimates of a new drug's efficacy as compared with a control substance that could be a placebo).These rules on preclinical testing may be relaxed for lifesaving treatments under the FDA's policy following the AIDs epidemic (National Academy of Sciences 1990).


Sometimes, surveillance studies are run, after the new product is marketed, to evaluate longer term safety and effectiveness. For some devices, many tests of a mechanism's function can be run in the laboratory and the effects of such devices in a human can more reliably be predicted than is the case for new drugs. For this reason, some implantable devices may be marketed without undergoing such exhaustive clinical testing. In the circumstances of a public health crisis, where a disease is both communicable and deadly, the use of pharmaceutical treatments is likely to offer the best approach, so I focus on them in the following discussion.

To develop a new drug from scratch and test it according to the usual rules, as already described, could take up to 15 years to reach fruition (Kaitin 2010). However efficient this process may be, many people might die before a new treatment could be fully tested, let alone subsequently made available to everyone. Moreover, the general chaos, public panic, decay of infrastructure, and health care worker sickness make it very difficult for us to control conditions sufficiently to allow robust research such as we might need to gather valid results. These research conditions could even be pragmatically incompatible with other measures used to control the spread of the disease.

That said, there are currently three main ways in which individual patients might try drugs as therapy in a pandemic. The first is under what is sometimes called the doctor's therapeutic privilege, but this legal justification relies on the drug in question having been tested for some other condition and having been licensed for this other particular use on grounds that it is safe and efficacious. The second is under compassionate or “named patient” access to a new drug. While interesting, we will see that the philosophical basis for justifying individual access to investigational treatments will not stand up to the circumstances of a public health crisis. The third is through the FDA's expedited or parallel track schemes, although direct or therapeutic access to the investigational treatment is still either delayed for most patients until after Phase II or denied if the patient is eligible for an ongoing conventional clinical trial.

Let us examine the therapeutic privilege. When faced with a severe threat to public health and many ill patients, much experimentation would probably be done by overworked general practitioners. These doctors might try cocktails of existing and licensed drugs. To count as a plausible treatment at all, there must be some rationale for trying it, with this rationale coming from inferential or analogous reasoning. A physician might infer from his or her theoretical knowledge of biochemistry or physiology that an existing drug might help in these new circumstances. For example, a doctor once tried Viagra on critically ill babies with respiratory problems, knowing that enlarging the blood vessels would increase oxygen supply to the brain (BBC 2007). Or any doctor, faced with a pandemic, might have previously observed beneficial clinical effects on similar symptoms in patients with similar conditions and then seek to apply this knowledge analogously. For example, neurologists and neuroscientists using deep brain stimulation (DBS) to treat Parkinson's disease noticed not only its effects on inhibiting movement but also its effects on mood (Scicurious 2012). Deep brain stimulation is now being tested for depression and other mental health conditions (Lozano et al. 2012). And indeed, the routine use of Viagra for male impotence was discovered accidentally by observing clinical effects in monkeys while testing it for angina (Terrett et al. 1996).

With the increased use of “personalized” medicine and treatment regimens that are based on particular genetic profiles, the scope for medical advance for everyone during a pandemic through the therapeutic privilege may be increasingly limited. These drugs would be licensed (and manufactured) for use in only small subpopulations of patients with the right genetic profile. Even if genetics did not preclude wider application, the drugs would be in short supply. Before we can concentrate on the challenge of manufacture on a grand scale, we must first explore further possibilities for science that seem to leave less to chance.

During a pandemic, for which little can be tested before the event, it is crucial to think how the regulatory restrictions on therapeutic access to new treatments might be relaxed in a way we might initially think is analogous to the compassionate access that is allowed by standard regulatory rules in an emergency situation. Where there is no threat to public health, some individuals are allowed access to experimental treatments on a compassionate and named patient basis, even if their safety and efficacy have not been fully demonstrated, provided that there is some preclinical information to support the rationale for their use (Edwards 2006; National Academy of Sciences 1991). The level of evidence required to provide lawful treatment may thus be lowered in some “exceptional” cases. The use of the word “exceptional” here is contentious even in these cases where a few individual patients who are very ill seek special prior and priority access before a marketing license has been granted for all similar patients. The word “exceptional” could refer to just one patient or it could refer to a single set but comprising several patients. I will continue to use the word for convenience only. In standard cases of an emergency, we thus accept that some individuals should have access to untested treatments on a compassionate basis. In view of the unusual needs of the community in a public health emergency, it might seem that we should simply extend therapeutic access to everyone in that community as they are all in the midst of an emergency. Under such circumstances, any regulatory restrictions on trying new drugs would then be so relaxed that they would disappear.

However, we cannot simply stretch the moral justification for treating a particular individual to the treatment of many individuals at a population level. This is because the rule of rescue, which we might use to support untested treatments for an individual, collapses as we introduce more, perhaps unidentifiable, individuals. In any case, the rule of rescue is far from being a normative rule and is more often discussed in the context of providing expensive care to an individual against the results of a cost–benefit analysis (Sheehan 2007). The rule of rescue could, at best, be thought to symbolize what a compassionate and humanitarian society might value *in extremis* under these financially restricted circumstances. Perhaps more usefully in the circumstances currently under consideration, that is, a pandemic, the rule of rescue could be thought to indicate when professional duties to individuals and to groups are clearly differentiated. For example, professional duties in public health and in intensive care are clearly distinguished in this way.^5^ More often, the rule of rescue reflects our psychological instinct to wade in and help the closest person who is in dire need of our assistance (Sheehan 2007). Interestingly, there is no law requiring us to intervene and rescue, except perhaps in France, mainly due to the inefficiencies such a rule would introduce (Posner 1981). We might find that no one takes on the role of health worker, for example, in order to avoid situations in which such a duty would require such a person to act.I thank Jonathan Wolff for helping me clarify this point in conversation.


Following the AIDS epidemic in the 1980s and complaints from patients with the disease, for which no treatment then existed, the FDA revised its policy on therapeutic access to investigational treatments (National Academy of Sciences 1991). During the development of zidovudine, for example, the FDA implemented policies to speed up the more restrictive trial phase. Interestingly, these are simply policies with no new regulation as such, since they were supposed to be compatible with existing rules. However, as we have seen, the philosophical justification for therapeutic access at an individual level is different from the philosophical justification for population-level restrictions on access to new medicines, which is principally to benefit patients in the future through gathering evidence. The particular FDA policy change of interest here, expedited development, is to remove the requirement for Phase III evaluation of drugs intended to treat life-threatening and severely debilitating diseases. Earlier trial phases, however, would then have to be designed with controls and probably extended in numbers to compensate for the absence of a separate Phase III. Importantly, therapeutic access would be restricted to research during the early phases. Further, postmarketing surveillance studies might also be required to supplement these data. While this means that patients as a group might have access to treatment sooner, expedited development does not solve the issue from an individual patient's perspective since the patient might still be too ill to wait for routine access after licensing, whenever that might be. Depending on the speed with which a pandemic were to take hold and the sheer number of patients in dire need of some treatment during these early phases of development, there may be many patients in the position of being denied therapeutic access because of these research constraints.

Similarly, the parallel track policy (again, developed by the FDA in response to the AIDS epidemic) makes selected investigational treatments available but only to HIV patients who are deemed ineligible for an ongoing conventional clinical trial. Under this policy, treatment outside research is allowed only once Phase II has been approved, and then only if Phase I provides expanded information (e.g., on different doses) and if patients can still be monitored for adverse effects.^6^ If eligible for a clinical trial, then a patient cannot simply choose to have the investigational treatment and so such treatment may be delayed. In a pandemic, many patients may die before Phase II is approved and so, again, be denied therapeutic access because of research constraints. In sum, the policies of both expedited and parallel track restrict access to investigational treatments in order to gain scientific knowledge for patients in the future. The philosophical justification for these policies was not made at the time when the new rules were adopted by the FDA, and for a pandemic I suggest they do not go far enough. This provides the motivation for further attention and regulatory work. To think about how regulators should respond to the special conditions of a pandemic, we must review the philosophy behind any restrictive regulation of investigational new drugs.Patients must be unable to participate in related clinical trials either because they do not meet the scientific eligibility criterion, perhaps on account of being too sick, or because their participation would create undue hardship.


## RESTRICTIVE REGULATION AND UNDUE CAUTION

Following public health problems, first with elixir sulfanilamide in 1937 and then with thalidomide in 1962, investigational new drugs have become increasingly regulated. It is important to note, though, that the drugs involved were not designed to treat deadly and communicable diseases. Implicit in all subsequent regulation (which restricts access to investigational new drugs to clinical trials to a greater or lesser extent) is the idea that we should use them only with caution, as there is scientific uncertainty about their safety. This philosophy rests on using the “precautionary principle.” The principle requires a certain level of evidence of safety before members of the public are exposed to threats of “serious or irreversible damage” (United Nations Environment Programme 1992). However, caution does not mean paralysis, as is made clear by the program's principle 15: “Lack of full scientific certainty shall not be used as a reason for postponing cost-effective measures to prevent environmental degradation” (UNEP 1992, Principle 15). This original description of the precautionary principle in the Rio Declaration has, however, since been changed to “when the scientific bases are insufficient or when there is *some uncertainty*” (EC, 2000, 23 [italics added]). As Holm and Harris (1999) observe, to require proof of safety before any new technology can be introduced does not permit scientific advance at all.

To accommodate this problem, Article 5.7 allows regulatory measures “where relevant scientific evidence is insufficient” to demonstrate the safety of a product (World Trade Organization [WTO] 1994, 72), but there is an obligation on regulators to take steps to obtain sufficient evidence. The account of the precautionary principle that was issued by participants at the 1998 Wingspread conference took this idea further and proposed that the burden of proof should not, however, lie with the regulators, although in theory it could. It should fall on the proponents of the activity to prove that it is safe, rather than on the regulators to prove that it is unsafe. While the precautionary principle instructs us to delay the introduction of potentially risky new practices in order to prevent harm, delay may itself cause harm in the form of lost opportunities to prevent disease and death (Harris and Holm 1999). The precautionary principle necessarily privileges an often indeterminate future risk over, possibly less apparent, current benefits. This difficulty was dramatically illustrated by the death of several thousand people in Peru in 1991 caused by removing chlorine from the municipal water supply after a risk assessment (by the U.S. Environmental Protection Agency) emphasized the calculated small risk from chlorination products but failed to address the greater risk of untreated water (Anderson 1991). Although the precautionary principle had little weight at that time, and was not explicitly used in the decision, this case is a clear illustration of the risks of its inappropriate application. Furthermore, in addition to downplaying any clear and present risks of not using new technologies, the precautionary principle ignores the possibility that future advances may reduce or eliminate any future risks.

The case of a pandemic might be an extreme example of this problem where threats of serious or irreversible damage are current and actual. The opportunity costs of restricting access to new technologies now in order to protect people in the future are clear: many people would die, and quickly. On closer inspection, the case of a pandemic might be special since the logic of the position already described does not withstand analysis. Future harm might not even be an empirical possibility. It is true that the human race has survived pandemics since we started living in close settlements and then towns and cities. We have survived the bubonic plague and deadly strains of influenza. Large genetic subpopulations of the human race, however, did not survive the Black Death (BBC World Service 2001). Our future as a species is in some sense contingent on how we act during a pandemic. There might be some chance of alleviating the threat through using technological advance, so any delay in gathering evidence could be catastrophic for some groups if not for everyone.

This is not to say that new medicines do not carry any risks at all, only that the benefits may be more apparent, given the alternatives, especially when there seems nothing or little to lose. Depending on how health states are valued, new drugs could do more harm than good even when individual patients are desperately ill and expect otherwise to die. I develop this point in the next section to show why some regulation of new medicines is necessary. For example, there are reports of patients with Alzheimer's disease (which is both serious and progressive) being worse off after having received embryonic stem cells (as part of research) than they would have been without any intervention (Freed et al. 2001). However, if we make the cost–benefit trade-off between certain death and the chance of a lingering life of poor health, the precautionary principle (which skews this trade-off against uncertain future harms) should itself be applied with caution. The precautionary principle, in general, serves as a useful check that any commercial interests are not served at the expense of public health, but it does not adjudicate between choices both of which are intended to promote public health. That said, it is worth noting that there are other precautionary measures available to the regulator, besides restricting new medicines to clinical trials, such as close monitoring and meticulous reporting to reduce uncertainty. Before exploring how clinical science could proceed in a pandemic (with due caution), I first establish why some regulation of investigational new drugs remains necessary.

## WHY SOME REGULATION IS STILL NECESSARY

Even under the extreme circumstances of a public health crisis and without appealing to the precautionary principle, it is evident that it might not, in fact, serve the public interest to allow would-be physician-researchers to try just anything to see where it leads, no matter what the cost–benefit calculation says for an individual patient. There are many reasons for this, but to stress that the thesis advanced in this article does not imply a wholly unregulated environment, I discuss the need for regulation based on experimental, current risk. As in the usual researcher–subject relationship, there remains an asymmetry of risk in favor of the researcher, which requires external oversight and supervision (Edwards 2009). These risks are associated with using treatments that produce unknown clinical effects. One reason for regulation of new medicines is that the threat of court action, through common law, is generally not sufficient to counteract fully the vested interests a researcher might have in his or her project, the pursuit of which might expose his or her subjects to greater risks (Edwards 2009). The most harmful outcome for the researcher is usually financial cost, loss of reputation or even a career, or even imprisonment (at worst). In the chaos of a pandemic, wayward researchers might never have to answer for malpractice. If the effects were serious enough, the subject could die from an experimental treatment.

However, where a disease is directly communicable, physician-scientists will themselves be on the front line for infection, and may place themselves at severe risk by seeing patients. It may be dangerous to perform humanitarian work without full infection control (as offered in clinics designed for such research work) requiring sterile environments, airlocks, and protective suits and masks.^7^ If the risk to the researcher is reduced by the use of special equipment, the subject will again be exposed to greater risks than the researcher (whose vested interests might remain without regulatory oversight). The preferential use of protective suits to protect health workers involved in research may raise questions of a political nature, which could, in turn, create social conflict and mistrust in the institution of research. However, if we were to accept arguments in favor of providing health workers with priority access to new therapies (so they are in a position to help treat many others in turn), they may themselves be the “subjects” of new treatments by virtue of being first.^8^
This was a worry during SARS, but it will be a more severe worry during a severe pandemic flu outbreak: “Even with excellent infection control practices, in the absence of vaccine, attack rates of greater than 10% are likely to occur among health care workers. Viral shedding of influenza occurs 1 to 2 days before symptoms are noted and can continue for 7 days after symptoms begin. Infants and immune-compromised individuals may shed for weeks, which makes transmission of influenza even more difficult to control both in the hospital and the community. In contrast, the Severe Acute Respiratory Syndrome (SARS) coronavirus shedding peaks at 7 to 10 days after symptoms begin, making this disease more easily contained with current infection control practices” (Cinti 2005, 63).
While I do not wish to be drawn into a discussion about the ethics of self-experimentation as such, it is perhaps enough to acknowledge that regulation is still important when there are unequal risks to researchers and subjects, whoever they might be.


Having shown why some regulatory oversight is necessary in conditions of a pandemic**,** I next explore how therapeutic access to investigational new drugs might be further expanded beyond the policies of expedited development or parallel track without abandoning the need for good evidence of safety.

## TOWARD NATURAL EXPERIMENTATION

Even given the luxury of a ready infrastructure and potential drug candidates, there seems little room for the full series of conventional clinical trials culminating in a randomized controlled trial. Such trials, while scientifically ideal in other circumstances to reduce selection bias, might be difficult or impossible to run in a pandemic. There are several reasons for this, each of which may be sufficient to make a conventional trial undesirable. First, as discussed earlier, it would take considerable time to complete formal testing even to Phase II, whatever level of statistical significance we were to accept (for speed) and whatever outcomes were measured (for convenience). Second, when no known treatment exists against which to compare a new therapy, any control substance would have to be a placebo. If a standard treatment is available but is known to be ineffective, then it technically counts as a placebo for the sake of therapeutic access to health care.^9^ However, though the topic is largely outside the scope of this article, it is noted that when effective treatments exist (and have been stockpiled, as in the case of pandemic flu) an active control could be used. In cases of individual emergencies where there is no communicable disease and where there is no alternative active therapy, placebo or ineffective treatment controls are indeed controversial. The problem stems from the possibility of a doctor denying a dying patient the last chance of benefit, which seems too cruel for physicians to countenance even when they are also scientists (Snowdon et al. 1997; Truog 1993; Worrall 2008). When a medically qualified researcher denies a patient a risky treatment that could work when the alternative is certain death, there is legal precedent to suggest that experimental treatment may be ethical and lawful (Edwards 2006). Indeed, the High Court in England ruled in favor of allowing doctors to try an experimental treatment (the drug pensotan polysulfate [PPS] injected directly into the brain) on a patient with variant Creutzfeldt–Jacob disease (vCJD; BBC 2002). For a physician to accept randomization, albeit in a blinded fashion, of the allocation of a placebo or a new treatment, the physician-researcher may already be violating duty of care as he or she is not in equipoise, and could simply select the new drug for the patient (or hedge as the HIV patients did in the corrupted AZT trials) to put the individual's interests first (National Academy of Sciences 1991). If, however, an effective standard treatment exists, then a conventional randomized controlled trial using an active control could be ethical, but the circumstances of a pandemic might make it impossible, as I will show. Third, we may not wish to put case and control subjects in close vicinity under the care of the same researcher for fear of further spreading the disease. Fourth, a conventional randomized trial is scientifically important only when the clinically worthwhile effect is moderate but nonetheless significant. In the case of a deadly pandemic, the outcome of interest would have very great significance, that is, many lives saved.Without perhaps any beneficial placebo effect.


It seems clear, therefore, that we need to rethink the design of research in these difficult circumstances in order to accommodate the need to provide as wide a therapeutic access to promising new treatments as possible (even beyond the immediate research population). We need somehow to combine the practices of research and routine care (which are otherwise thought to be in contrast or even to be mutually exclusive, given standard regulatory restrictions on access). Observational work, or natural experiments in the field, may become more important. A simple anecdote about the successful treatment of one patient might be enough to warrant trying it on another, and so on, until there are enough observational data available to justify more widespread use. As many patients could be dying, without treatment, any reversal of fortune would suggest, if not establish, a dramatic effect and so form a scientific rationale for further investigation. With more lives saved, confidence in the new drug would increase. Once a new drug appears to work and it is available to many patients, then more robust observational data might be collected and pooled.^10^ The best information in these conditions may be far from the clean aggregate data from a conventional randomized controlled trial and may start as a string of promising anecdotes or a “case series.”Note that a dramatic effect would not, in any case, require a large randomized controlled trial to become apparent. For example, we did not need a randomized trial of aspirin to observe its large clinical effects. Randomized trials are usually only ever a epistemic necessity when seeking moderate yet worthwhile effects.


Manufacturing a sufficient quantity of any new drug might itself take time, so the drug may become available for distribution only gradually, allowing researchers and doctors to adapt their approach to their accumulating clinical observations. At any point, another new treatment might be identified or the drug currently being distributed might have to be discarded (either because of severe side effects or because of apparent inefficacy) or the recommended dose might change quickly. Natural experiments of many different drugs simultaneously may be based on a quasi-random allocation with a flexible analysis. In this vein, Bayesian techniques might be better able than alternative statistics to cope with analyzing rapidly changing technologies and providing meaningful data (Lilford et al. 2000). Comparing regimens in a changing and chaotic environment will be essential.

Cooperative methods for trying out new treatments and for sharing epidemiological and effectiveness data seem crucial (Langat et al. 2011). However, the prospects for sharing data between countries may be more limited than we might imagine, even with coordination provided by the WHO.^11^ A lot of the information flows informally, but the formal sharing of confidential documents has previously proved impossible. With time, there might be formal agreements in place to facilitate sharing.Professor Neil Ferguson (Director, MRC Centre for Outbreak Analysis and Modelling) warned the UK Science and Technology Committee for Parliament that “while Governments and countries are happy to share analysis—their view of the situation—they are rarely willing to share the detailed data they are collecting in real time, or at least some of it. … We had very detailed data from the US CDC, data from Mexico and other countries. We couldn't share it with the other partners we were working with. We could only share a kind of synthesis. … It was not so much of an issue last year because it was relatively mild, but there were instances where, had we been dealing with something more serious, it could have posed some problems and we could have lost some efficiency about that inability to share raw data.”


Any regulatory decisions based on the precautionary principle would thus be provisional, which means that they should be kept under active review and modified when further information that reduces uncertainty becomes available. At the beginning of a possible outbreak, politicians may not realize the full extent of the problem and may be reluctant to take draconian measures. With novel diseases, all relevant nonpharmaceutical precautions may be taken first until there is sufficient evidence to allow their relaxation. Here, the precautionary principle might refer to social organisation rather than the use of new technologies. For this purpose, health workers and law enforcement agencies need to understand the scale of the problem through social control and basic surveillance. By tracking the incidence of cases, they will learn about the mechanism by which the infection is spread and how quickly it takes hold. The need to stop the spread of a new pathogen might persuade researchers and politicians to restrict freedom of movement. International travel associated with affected regions might be shut down (which is what the WHO recommended during the SARS outbreak in Toronto, Canada, much to the distress of the politicians concerned with maintaining international trade and protecting their economy). In some cases, physical containment is still the only way to manage a public health emergency. Ebola, for example, is confronted simply by isolating the community affected and allowing the disease to run its course (Calain 2009).

With these two considerations in mind, controlled research (as far as it might be possible and ethical in a pandemic) might be advanced by using wedged cluster designs (Edwards et al. 1999; Edwards et al. 2012). A new investigational vaccine or other treatment would be introduced to selected groups at a chosen time with those waiting for the treatment acting as control groups. As more of the drug is manufactured, groups acting temporarily as control groups could be included in the study. Some groups will not receive the treatment immediately and, as I will show, the process by which the groups are selected and then assigned to treatment raises issues of fairness.

For ease of delivery, allocation of new medicines is usually then determined by geography or service supply. New treatments could be delivered more efficiently to larger numbers through existing infrastructure and routine services such as a water supply, although this is contentious as it draws traditional ideas of research ethics such as informed consent into question (Edwards et al. 1999; Sabin et al. 2008). There is not the same issue of consent in individual-cluster trials where the treatment is given separately to each individual once the relevant cluster has been assigned to a trial arm (Edwards et al. 1999).

In this way, it is possible to achieve as wide a therapeutic access as possible while collecting research data. The results obtained by such trials reflect the treatment's safety and effectiveness (which is the treatment's effects under real-world conditions) rather than its efficacy (which is the treatment's effects under the artificial laboratory conditions, simply to establish cause and effect). To reduce the scientific issue of selection bias in allocation, it is possible to randomize the intervention to clusters (CRCTs).

Furthermore, cluster trials, whether randomized controlled or small pilots, fit well with other nonpharmaceutical strategies in a pandemic. While there is very little robust evidence on the effectiveness of nonpharmaceutical interventions themselves for the control of public health emergencies, they will prove invaluable and necessary for the purpose of gathering controlled data about the effects of pharmacological advances. The collection of high-quality clustered data for research may thus be a useful side effect of geographical containment. In this way, major nonpharmaceutical strategies (those of physical and hence social distancing or isolation) could enhance our capacity for pharmacological advances. Once the problem is recognized, and in the absence of epidemiological knowledge, the first defense is quarantine. This might seem pragmatically easy since patients who are very ill will be confined in intensive care.

In addition, physicians’ professional duty of care to each individual patient can remain intact and they can follow the rule of rescue, as described earlier, without compromising the wider public health effort. With cluster designs, there will be no single physician-scientist handing out a drug to one patient and then refusing to treat the next in line (Sabin et al. 2008).

We might argue that more, rather than less, evidence of safety is required before the cluster trial starts, as more people would be affected at once and in a less controlled environment. However, each cluster could contain as small a number of individuals as allowed by the methodological techniques and as advisable in light of the preclinical evidence, and individual subjects within each cluster could be recruited sequentially to manage the risk as well as is possible in the circumstances. Such an approach might sound very much like a traditional Phase I trial. However, the crucial difference is that individuals are recruited from predefined clusters rather than the general patient population. There is no methodological reason why clusters should not comprise different numbers of individuals. Indeed, there are already recognized methods that could incorporate arbitrary clusters, for example, using generalized estimating equations (GEE) or using a robust and automatic calculation of standard errors in STATA.^12^
I thank David Spiegelhalter for his insights on incorporating arbitrary clusters.


After the crisis has been averted, it will also be important to monitor the effects of any new drugs that have been distributed. This will be a form of longer term surveillance akin to the study of ill health after natural disasters or commercial accidents, such as a leak from a nuclear power plant. Such surveillance is easier when directed at a community or population rather than at diverse and disparately located individuals. For example, it has only been through longer term surveillance following the swine flu scare of 2009/2010 that the questionable effects of the “swine flu jab” (Pandemrix) have come to light (CDC 2010). Links to rare cases of narcolepsy, a disorder that causes people to fall asleep suddenly and unexpectedly, have been reported, mainly from Finland and Sweden but also from Iceland and the United Kingdom. A nonrandomized study of children, some of whom had been vaccinated with Pandemrix, suggested that it carries a 6- to 13-fold increase in risk of narcolepsy (CDC 2010). Overall, it has now been given to more than 31 million people worldwide and, because of the potential seriousness of the H1N1 infection, Pandemrix remains a licensed alternative for children (at the time of writing). At least, however, the effects have been reported, so further work can be organized through the medicines regulators. This was not so in the wake of the SARS outbreak. “After the SARS outbreak in 2003, thousands of patients were treated with agents of unproven efficacy and definite toxicity; data on these agents’ efficacy were not gathered. To prevent this situation from repeating itself, we must be prepared to conduct prospective, randomized controlled trials in the event of future outbreaks of novel pathogens” (Muller et al. 2004). While the appeal to gather surveillance data is laudable, the randomized trials to which Muller refers may be feasible and, arguably, ethical only using cluster designs.

However, there may be moral and political concerns about the use of cluster designs, concerns that could, if mounted without careful forethought and governance, be exacerbated by features of the pandemic itself. This is due to resultant inequalities or commercial exploitation, or both.

## TRUST AND FAIRNESS IN CLUSTER TRIALS

In nonemergency circumstances there are potential problems with using randomized cluster designs in which clusters are bounded by socially divisive fault lines (Conrad and Edwards 2011), and any risks associated with consequent social tensions are only amplified by the special features of a public health emergency (London 2011). It is particularly important to maintain the community's trust in social institutions during a pandemic if there is to be any semblance of law and social order. However, two aspects of testing new drugs using CRCTs might erode this trust. One problem, exacerbating inequalities, is inherent to the design itself, while the other, profiteering, is only associated with involving industry. Financial exploitation is not the only problematic form of exploitation possible, but we can nevertheless usefully discuss it in isolation, since it could be subject to a form of governance that could be organized in advance of a pandemic. In the last analysis, abuse of political power may only be resolved, in practice, after the event when governments are brought to account for their actions. With commercial interests at play, it is not obvious how industry can legitimately be involved without inviting exploitative practice but there is arguably greater scope for prior analysis and governance by government itself.

### Group Inequalities

We have seen that people are divided into groups, prior to any consideration of research, in order to contain the disease within defined geographical areas. However, clusters formed for the purposes of running controlled research would have to be compatible with strategies for social isolation. Depending on how local communities are formed, providing access to promising new therapies sequentially according to geographically defined clusters may exacerbate any preexisting social inequalities.

Many major theories in political philosophy consider inequalities to be bad in themselves, and focus either on avoiding them (by strategies such as egalitarianism) or on giving special priority to worse-off groups (using techniques such as prioritarianism). Inequalities may also lead to other bad outcomes, including mistrust in government or prejudice (Uslaner and Brown, 2005; Wilkinson and Pickett, 2006).

Randomization has, in some cases, been used first as a fair method of distributing scarce resources in nonemergency conditions and as a method for adding scientifically valuable research (Edwards and Kirchin 2002). But CRCTs could exacerbate existing health inequalities if they are not subject to genuine random allocation of the new drug. If there is good reason to believe that the group receiving the new treatment first would fare better than the group acting as control, those with political power would have good reason to rig the randomization process to make sure their constituents get the treatment first. This also makes the exacerbation of preexisting inequalities more likely, because the population of more affluent districts might well be more politically active, consequently having more political influence over the allocation process.

However, we would not necessarily expect, before the trial, that any one arm would fare better than another (Freedman 1987). This is because under conditions of equipoise, we do not know whether the trial intervention is better or worse than the control intervention. We could, however, argue that we should not require equipoise in randomized cluster trials. The requirement of equipoise is always very demanding, even in the context of conventional RCTs. Scientists must have some reason for thinking that the new treatment is superior to currently accepted treatments; otherwise there would be no rationale for testing and there would be no basis for calculating the necessary sample size. The larger the cluster trial, the more resource and organization it would take to coordinate and, we might think, the greater the belief in the new treatment required to pursue it, moving the investigators further from the state of equipoise.

Furthermore, we would require larger numbers of clusters or larger expected treatment effects to gain adequate statistical power. Estimating the sample size needed to show a significant difference is complicated in cluster designs by an intra-class correlation (measured by the intraclass correlation coefficient [ICC]) (Ukoumunne et al. 1999). This is due to the fact that the individuals belong to the same cluster or group, defined by social or geographical boundaries. They are likely to exhibit a degree of homogeneity, which may influence the measure studied in the trial. Indeed, the between-cluster variation will sometimes be larger than the variation between individuals within a cluster. To achieve the desired statistical power, scientists could increase the total number of clusters (Ukoumunne et al. 1999), or they could choose to test only those treatments for which the anticipated effect is relatively large. By increasing the number of clusters we would inevitably increase the total number of human participants, which, in turn, might have implications for fairness. This would subject a greater number of people to any trial-generated inequality. Increasing the threshold for an intervention effect would increase the size of the generated inequality. In a virulent and deadly pandemic, we would be looking for the largest effect size possible.

To compound these problems, the perception of group inequalities is likely to be worse, especially in a pandemic. Cluster trials may compound any issues of a “postcode lottery” nature, where different local districts implement different substantive policies (Devlin 2008). They are unlikely to form a new devolved political structure, which results in a postcode lottery of treatment specifically for the disease in a pandemic. This is because the delivery of the new treatment will be determined by preexisting structures that govern the development of strategies of geographical containment. However, a CRCT that was centrally planned (e.g., by the World Health Organization [WHO] as the directing authority for health within the United Nations) would not be able to justify differentially implemented policies in order to protect a greater right to the autonomy of individual districts. One may appeal to autonomy to protect the right of or group leaders to enroll their communities in research, but not to protect the right of scientists to plan a cluster trial centrally. If well-publicized cluster RCTs were seen as socially unacceptable, this could hamper the entire public health effort.

Human psychology, moreover, shows that the perception of risk is influenced by clustering biases. When asked in several studies to compare different risky technologies, lay people judged the same number of fatalities as more serious when caused by a single accident (harming groups) than when they are dispersed across several events (harming isolated individuals) (Vlek and Stallen 1981; Slovic et al. 1979; Slovic et al. 1980). More recently, Slovic and colleagues showed that clustered risks from a single event are particularly problematic when the probabilities are largely imprecise while exposure to those risks is uncontrollable and potentially catastrophic (Slovic et al. 1984). These clustered risks, however, can be made more acceptable with greater anticipated benefits, especially to those clusters exposed to the risks (Vlek and Stallen 1981). For these reasons, a cluster trial of a promising new treatment may be seen as acceptable on the grounds that the same group exposed to the risk of the treatment also stands to benefit. However, it is more likely that, in a pandemic, the risks of not receiving anything would be seen to be problematic especially when the effects of any inequality would be long-lasting or when they would be impossible to address afterwards (or through the sequential delivery of treatment by cluster). By the time the new treatment has been fully rolled out, many in some acting control (allocated to delayed treatment) clusters may have died. However, the number of deaths is likely to be far fewer than if the treatment were first restricted to research, and only then rolled out to the wider population.

In sum, as one of the main points behind my thesis is that research data can be collected, and processed, while rolling out a new treatment to the population at large (rather than artificially restricting access to it until after the results are obtained through research, as currently regulated), it is difficult to say that clusters should be selected to match socioeconomic factors to avoid generating inequalities within the trial. Those not selected as eligible would lose out. Rather, the allocation of clusters, whatever their social status, could be randomized if the process were genuine, without political perversion, and there were sufficient epistemic uncertainty surrounding its effects. In this way, members of the public may hold sufficient trust in the research to accept it. The degree of public trust could be assessed, hypothetically, in advance; there are already mechanisms for gauging public opinion about medical research generally (National Institutes of Health [NIH] 2005).

As soon as these drugs show promise and are evaluated, however, the issue of manufacture comes to the fore and relationships with industry will need to be negotiated, assuming that any commercial manufacturing facilities are not simply seized by government. The inescapable issue of fairness discussed earlier could then be grossly compounded by commercial exploitation. These relationships are less easy to conceptualize but they could be governed, in the event of a pandemic, by government.

### Commercial Interests

While the effect of commercial involvement is a consideration that is not an integral part of research in a pandemic, assessment of its capacity to erode public trust in a situation where trust is needed and commercial involvement useful (if governed robustly) is a good (and obvious) place to start. After the swine flu pandemic of 2009/2010, Dame Deidre Hine was commissioned to review the UK government's response and specifically to examine the strategies adopted by the devolved administrations. There had been more than 800,000 cases of swine flu across the United Kingdom and 457 deaths, including those of children and pregnant women. The review put the total cost of the pandemic at £1.2 billion, taking into account both the preparation and response stages. The vast proportion of that total, around £1 billion, went to pharmaceuticals, which included the antiviral drug Tamiflu (the vaccine for swine flu) and antibiotics.

While the review concluded that there was no overreaction, it recommended that lessons be learned and showed us that industry can drive a hard bargain with government even in the midst of a public health crisis. One of those lessons, for example, was that the UK Department of Health should have negotiated a “break clause” in its contract with drug company GlaxoSmithKline, to allow drug and vaccine supply to be tailored to need. There was initially flexibility in the amount the United Kingdom could purchase, ranging from 30 million doses to 132 million doses (which was enough to vaccinate the whole UK population with two doses). However, once contracts had been signed, the full consignment was purchased regardless of evolving need.

There are signs, however, that the power of the pharmaceutical giants is generally on the wane. Interestingly, part of the U.S. National Institutes of Health investment for translational medicine is in basic infrastructure to enable the closer collaboration of those researchers thought important to the project. However, while government is funding institutes for translational medicine, the pharmaceutical industries are closing their large research and development facilities after several years with disappointing results. The next big blockbusters, they suggest, will come from smaller, more concentrated, groups. This may give public authorities greater negotiating power when facilities are needed to increase the pace of research in a pandemic. An example of the reduced power of pharmaceutical companies is the action of the Indonesian government, which refused to hand over samples of the H5N1 avian influenza virus to the WHO in 2007 unless its citizens were guaranteed access to any resulting vaccine, thereby creating a global health governance crisis (Fidler 2008). And the Indian courts have recently issued a compulsory license or treatment order that effectively defeats any patent advantage a company might use to charge above what developing countries can afford for treatments (Global Health Check 2012).

## CONCLUSION

As the policies of social containment and drug discovery are applied in a public health emergency, I have suggested that regulators should consider ways in which access to investigational new drugs could be expanded beyond the policies laid out by the FDA after the AIDS epidemic in the 1980s. With the emphasis on nonpharmaceutical methods of geographical and social containment and on what observational or natural experiments are compatible with them, wider access to new drugs may not mean losing scientific evidence of safety. For example, cluster trials could provide a way to evaluate new drugs and gather meaningful scientific data while managing the spread of disease and the wider distribution of new drugs. The effects of the new treatments could be evaluated while they are being rolled out to the population. Yet concern for fairness requires any research be planned with ensuing group inequalities and commercial exploitation in mind. Genuine randomization of clusters and robust political systems help to address the inescapable issue of fairness, while those with a duty of care to individual patients are protected by policy. Commercial interests should, to a large extent, be governed by government. While we are free of the panic of a pandemic, it would be wise to think through these questions, consider the views of the public, and prepare policy for such an eventuality.
